# The small organic molecule C19 binds and strengthens the KRAS4b-PDEδ complex and inhibits growth of colorectal cancer cells in vitro and in vivo

**DOI:** 10.1186/s12885-018-4968-3

**Published:** 2018-11-01

**Authors:** Pedro Cruz-Nova, Michael Schnoor, José Correa-Basurto, Martiniano Bello, Paola Briseño-Diaz, Arturo Rojo-Domínguez, Carlos M. Ortiz-Mendoza, Jorge Guerrero-Aguirre, Francisco J. García-Vázquez, Rosaura Hernández-Rivas, María del Rocío Thompson-Bonilla, Miguel Vargas

**Affiliations:** 1 Departamento de Biomedicina Molecular, Centro de Investigación y de Estudios Avanzados del Instituto Politécnico Nacional (CINVESTAV-IPN), Av. I.P.N, 2508 México City, Mexico; 2 Laboratorio de Modelado Molecular y diseño de fármacos de la Escuela Superior de Medicina, Instituto Politécnico Nacional, México City, Mexico; 3 Departamento de Ciencias Naturales, Universidad Autónoma Metropolitana Unidad Cuajimalpa, México City, Mexico; 4 Investigación Biomédica y Traslacional, Laboratorio de Medicina Genómica, Hospital 1° de Octubre, ISSSTE, México City, Mexico; 5 Laboratorio de inmunohistoquímica, Instituto Nacional de Pediatría, México City, Mexico

**Keywords:** Colorectal cancer, KRAS4b, PDEδ, Apoptosis, Erk, Akt

## Abstract

**Background:**

Colorectal cancer is the third most common cancer worldwide; and in 40% of all cases, KRAS4b-activating mutations occur. KRAS4b is transported by phosphodiesterase-6δ (PDEδ) to the plasma membrane, where it gets activated. PDEδ downregulation prevents redistribution and activation of KRAS4b. Thus, targeting the KRAS4b-PDEδ complex is a treatment strategy for colorectal cancer.

**Methods:**

Using docking and molecular dynamics simulations coupled to molecular mechanics, the generalized born model and solvent accessibility (MMGBSA) approach to explore protein-ligand stability, we found that the compound ((2S)-N-(2,5-diclorofenil)-2-[(3,4-dimetoxifenil)metilamino]-propanamida), termed C19, bound and stabilized the KRAS4b-PDEδ complex. We investigated whether C19 decreases the viability and proliferation of colorectal cancer cells, in addition to knowing the type of cell death that it causes and if C19 decreases the activation of KRAS4b and their effectors.

**Results:**

C19 showed high cytotoxicity in the colorectal cancer cell lines HCT116 and LoVo, with a stronger effect in KRAS-dependent LoVo cells. Importantly, C19 significantly decreased tumor size in a xenograft mouse model and showed lower side effects than 5-fluorouracil that is currently used as colorectal cancer treatment.

**Conclusions:**

Mechanistically, the cytotoxic effect was due to increased apoptosis of tumor cells and decreased phosphorylation of Erk and Akt. Therefore, our results suggest that C19 may serve as a promising new treatment for colorectal cancer.

**Electronic supplementary material:**

The online version of this article (10.1186/s12885-018-4968-3) contains supplementary material, which is available to authorized users.

## Background

Colorectal cancer (CRC) is the third most common cancer worldwide, and the second most common cancer regarding the number of individuals surviving five years after diagnosis [1]. Approximately, 40% of colorectal cancer cases have been associated with single base missense mutations in the small GTPase KRAS4b [2]. KRAS4b acts as a central relay for signals originating at receptor tyrosine kinases such as the EGFR family in the intestinal epithelium and in many other tissues. Most mutations lock KRAS4b in the GTP-bound conformation causing constitutive activation and binding to effector molecules such as RAF kinases [3]. To get physiologically activated, KRAS4b must be translocated from the endoplasmic reticulum to the plasma membrane; a process mediated by phosphodiesterase 6δ (PDEδ) which is a solubilizing factor-like guanylate dissociation inhibitor (GDI). The farnesyl group in the C-terminal region of KRAS4b promotes anchorage to the plasma membrane [4, 5]. PDEδ levels and oncogenic mutations determine the activation of KRAS4b after growth factor receptor stimulation. Given that KRAS4b mutations are associated with treatment-resistant tumors in metastatic colorectal cancer [6, 7], the KRAS4b-PDEδ complex has been suggested as a potential target for the generation of new cancer therapeutics [4]. Fluoropyrimidines, oxaliplatin and irinotecan are common chemotherapeutics for metastatic colorectal cancer, but their sequential administration only results in median overall survival ranging from 18 to 20 months [8]. The development of targeted therapies in colorectal cancer with mutated KRAS4b has failed thus far in the clinic [2].

To identify specific inhibitors of oncogenic KRAS4b signaling in pancreatic cancer, we recently discovered by bioinformatical screening 38 new small molecules that bind and stabilize the KRAS4b-PDEδ complex [9]. However, only three of these compounds decreased total RAS activation in vitro and had a specific cytotoxic effect in pancreatic cancer cells. Importantly, these compounds showed low cytotoxicity in normal pancreatic cells. In a murine xenograft tumor model, one of these small molecules, (N-[(2H-1,3-benzodioxol-5-yl)methyl]-2-[4-(5-chloro-6-oxo-1-phenyl-1,6-dihydropyridazin-4 yl)piperazin-1-yl]acetamide)), termed D14, remarkably decreased tumor size and triggered apoptosis of tumor cells. Following the same approach, we analyzed whether these molecules also have cytotoxic activity in colorectal cancer cells with mutated KRAS4b.

In this work, we report that the compound ((2S)-N-(2, 5-diclorofenil)-2-[(3, 4-dimetoxifenil)-metilamino] propanamide) termed C19 binds and stabilizes the KRAS4b-PDEδ complex. Moreover, C19 has specific cytotoxic activity in colorectal cancer cells with oncogenic KRAS4b mutations in vitro and in vivo by decreasing proliferation, promoting apoptosis and preventing phosphorylation of Erk and Akt. Thus, C19 could be a promising candidate for new treatment strategies in colorectal cancer.

## Methods

### In silico docking simulations

We identified the chemical structure of C19 using the ENAMINE database 3D *Diversity set* (www.enamine.net) by docking to the interface region of the molecular complex KRAS4b-PDE**δ** using AutoDock 4.2.626 [10] and MOE Dock (www.chemcomp.com). The coupling calculations were carried out using the recommended standard parameter settings. We evaluated a maximum of 250,000 poses for C19 on the receptor target (crystallographic contacts between KRAS4b with PDE**δ**, from PDB ID 5TAR). Grids were calculated using Autogrid 4.2.626 [10] with a spacing of 0.375 Å by focusing on the interface of the crystallized proteins. Molecular docking with MOE 2014.09 [11] was performed using the matcher function to generate the initial poses. The best 30 results from the London dG score were further refined using energy minimization with MMFF94x force field and rescored using Affinity dG scoring.

### Molecular dynamics simulation

MD simulations of the protein-ligand complex were performed using AMBER 16 package [12] and the ff14SB force field [13]. Ligand charges for non-parameterized residues in proteins were determined using the AM1-BCC level and the general Amber force field (GAFF) [14] For protein-ligand complex, a 15 Å rectangular-shaped box of the TIP3P water model [15] was applied to solvate the complex; and Cl^−^ and Na^+^ ions for the protein-ligand system were placed in the model to neutralize the positive or negative charges around the complex at pH 7. Before MD simulation, the system was minimized through 3000 steps of steepest descent minimization followed by 3000 steps of conjugate gradient minimization. Then, the system was heated from 0 to 310 K during 500 ps (ps) of MD with position restraints under an NVT ensemble. Succeeding isothermal isobaric ensemble (NPT) of MD was carried out for 500 ps to adjust the solvent density followed by 600 ps of constant pressure equilibration at 310 K using the SHAKE algorithm [16] on hydrogen atoms and Langevin dynamics for temperature control. Equilibration run was followed by 100 ns-long MD simulation without position restraints under periodic boundary conditions using an NPT ensemble at 310 K. The particle mesh Ewald method was utilized to describe the electrostatic term [17], and a 10 Å cut-off was used for van der Waals interactions. Temperature and pressure were preserved using the weak-coupling algorithm [18] with coupling constants τ_T_ and τ_P_ of 1.0 and 0.2 ps, respectively (310 K, 1 atm). The time of the MD simulation was set to 2.0 femtoseconds, and the SHAKE algorithm [16] was used to constrain bond lengths at their equilibrium values. Coordinates were saved for analyses every 50 ps. AmberTools14 was used to examine the time-dependence of the root mean squared deviation (RMSD), radius of gyration (R_G_), and clustering analysis to identify the most populated conformations during the equilibrated simulation time.

### Calculation of free binding energies

Calculation of free binding energies was carried out using the MMGBSA approach [19–21] provided in the Amber16 suite [12] 500 snapshots were chosen at time intervals of 100 ps from the last 50 ns of MD simulation using a salt concentration of 0.1 M and the Generalized Born (GB) implicit solvent model [22] The free binding energy of the protein-ligand system was determined as follows:

$$ \Delta {G}_{bind}={G}^{complex}-{G}^{receptor}-{G}^{ligana} $$

$$ \Delta {G}_{bind}=\Delta {E}_{MM}+\Delta {G}_{solvation}-T\Delta S $$

*ΔE*_*MM*_ represents the total energy of the molecular mechanical force field that includes the electrostatic (Δ*E*_*ele*_) and van der Waals (Δ*E*_*vdw*_) interaction energies. Δ*G*_*solvation*_ is the free desolvation energy price upon complex formation estimated from the GB implicit model and solvent-accessible surface area (SASA) calculations yielding Δ*G*_*ele,sol*_ and Δ*G*_*npol,sol*_. *TΔS* is the solute entropy arising from structural changes that occur in the degrees of freedom of the free solutes when forming the protein-ligand complex.

### Cell culture

All cell lines were purchased from ATCC (Manassas, VA). Human colorectal cancer cell lines HCT116 (ATCC® CCL-247™) and LoVo (ATCC® CCL-229™) were cultured in RPMI-1640 Medium (ATCC® 30–2001™) and F12 K medium (Kaighn’s Modification of Ham’s F-12 Medium) (ATCC® 30–2004™) (ATCC Manassas, VA), respectively. The non-cancerogenic human colorectal cell line CCD-18Co was cultured in Eagle’s Minimum Essential Medium (EMEM) (ATCC® 30–2003™) (ATCC Manassas, VA). All media were supplemented with 10% fetal bovine serum (FBS) and 1% penicillin/streptomycin solution. Cells were cultured in a 5% CO_2_ atmosphere at 37 °C.

### Cell viability

HCT116, LoVo and CCD-18Co cell lines were plated in 96-well plates at a density of 1 × 10^4^ cells/well and grown for 24 h. Cells were then treated with C19 for 72 h in complete medium. To assess viability, cells were analyzed using the CellTiter-Glo kit (Promega, Madison, WI) according to the manufacturer’s instructions. The concentration of C19 that killed 50% of all cells after 72 h (IC_50_) was determined by applying curve-fitting analyses with Prism software (GraphPad Software, San Diego, CA, USA).

### Clonogenic assay

Colorectal cancer cell lines were seeded in 6-well plates at a density of 300–500 cells per well and cultured overnight. HCT116 and LoVo cells were treated with final concentrations of 5 and 10 μM of 5-fluorouracil (5-FU; PiSA Laboratories, México), respectively, and the IC_50_-concentrations of C19 for 72 h. Subsequently the medium was replaced with fresh medium supplemented as mentioned above for a total duration of 10 days. Cells were then fixed with 4% paraformaldehyde (PFA) at room temperature for 10 min and washed with distilled water. Cells were stained with 0.1% crystal violet in water for 30 min, washed with water, dried, and photographed. For quantification, 10% acetic acid was added for 5 min to extract the dye and the absorbance was measured photometrically at 590 nm using a TECAN Fluorometer Infinite F500 (Tecan Austria GmbH).

### Flow cytometry

Apoptosis was detected using the FITC-Annexin-V/dead cell apoptosis kit (Invitrogen) according to the manufacturer’s protocol. Briefly, cells were seeded at a density of 5x10^5^cells per well in six-well plates and cultured with the IC_50_-concentration of C19 for 72 h. Untreated cells served as controls. Cells were trypsinized, washed with ice-cold PBS and suspended in 1x binding buffer (50 mM HEPES, 700 mM NaCl, 12.5 mM CaCl_2_, pH 7.4) at a concentration of 1 × 10^6^ cells/mL. 5 μl of FITC-Annexin-V solution (25 mM HEPES, 140 mM NaCl, 1 mM EDTA, pH 7.4, 0.1% bovine serum albumin) and 1 μL of Propidium iodine (PI) solution (100 μg/ml) were added to 100 μL of each cell suspension. Cells were then gently vortexed and incubated at room temperature in the dark for 15 min. Then, 400 μL of ice-cold binding buffer was added and gently mixed. Fluorescent intensities were examined by flow cytometry using a FACS Calibur cytometer (BD Biosciences, San Jose, CA) followed by data analysis using FlowJo software (Tree Star Inc., Ashland, OR).

### RAS-GTP pull-down assay

The RAS-GTP pull-down assay was performed using the RAS Activation Assay Biochem Kit (BK008, Cystoskeleton, Inc. Denver, CO), according to the manufacturer’s protocol. Briefly, HCT116 and LoVo cells were grown to 80% confluence (~ 3 × 10^6^ cells) in 100-mm tissue culture dishes and lysed in 400 μL of ice-cold lysis buffer supplemented with protease and phosphatase inhibitors. Lysates were cleared by centrifugation, and 300 μg protein from each sample was collected. Lysates were incubated by end-over-end rotation with 100 μg Raf-RBD-conjugated beads for 1 h. The supernatant was then carefully removed and the beads were washed and boiled in 2X Laemmli sample buffer, followed by Western blot analysis which was performed using the pan-RAS antibody provided with the kit.

### MAPK activation profiling

Cells were rinsed with cold PBS and immediately lysed in buffer supplemented with 4xcOmplete™ EDTA-free Ultra Protease Inhibitor Cocktail (Sigma-Aldrich) and 1xPhosSTOP™ (Sigma-Aldrich) at 4 °C for 30 min. Following centrifugation at 14,000×g for 5 min, supernatants were transferred into a clean tube and protein concentrations were determined using the Precision Red Advanced Protein Assay (Cytoskeleton, Inc. ADV02-A). Lysates were diluted and analyzed using the Human Phospho-MAPK Arrays (Proteome Profiler; R&D Systems; Minneapolis, MN, USA) according to the manufacturer’s instructions. Nitrocellulose membranes were scanned using a ChemiDoc™ Imaging Systems (BIO-RAD Laboratories, Inc.).

### Xenograft model: Subcutaneous injection and pre-clinical drug testing

All animal experiments have been approved by the institutional animal care and use committee of Cinvestav (Protocol Number 0238–17). Animals were housed in the animal facility of Cinvestav (Mexico-City, Mexico) under standard conditions. Six-week-old nu/nu male mice with a weight of approximately 20 g were used. 5 × 10^6^ LoVo cells were resuspended in 200 μL PBS and injected subcutaneously in the right flanks of nude mice. When tumors reached an average volume of 150 mm^3^, mice were randomly assigned into three groups: vehicle only (10% DMSO in 1x PBS); C19 (15 mg/kg and 30 mg/kg), and 5-Fu (10 mg/kg). Treatments were administered intraperitoneally in a final volume of 300 μl once daily for 12 days. Tumor volumes were calculated using the formula: mm^3^ = dxD^2^/2, with d and D being the shortest and longest diameters, respectively.

### Western blots

Tissue samples were weighed, snap-frozen, and crushed in liquid nitrogen in a mortar. Samples were transferred to a microcentrifuge tube and lysed using ProteoJET™ Mammalian Cell Lysis Reagent. After centrifugation at 20,000 x g for 15 min and protein quantification, SDS-PAGE was carried out using 30 μg protein of each sample. Proteins were transferred onto PVDF membranes (Merck Millipore) and blocked for 1 h at room temperature using PBS containing 5% skim milk. Antibodies used for western blotting were total Erk (Cell signaling-9102; 1:1,000), pErk (Cell Signaling-9101; 1:1,000), total Akt (Cell Signaling-9272 1:1,000), pAkt (Cell Signaling-4060 1:500), PARP (Cell Signaling-9542 1:500), PCNA (Zymed 13–390 1:500) and β-Actin (GeneTex, 1:500). Primary antibodies were incubated overnight at 4 °C and washed off. Species-specific HRP-conjugated secondary antibodies were then incubated for 1 h at room temperature and washed extensively. Membranes were incubated with Super Signal West Femto substrate (Thermo Fisher Scientific) and signals were recorded using a ChemiDoc™ Imaging Systems (BIO-RAD Laboratories, Inc.).

### Immunohistochemistry

Paraffin sections of colon tumor samples were deparaffinized in xylene and rehydrated in a series of graded alcohols. Antigens were retrieved in 0.01 M sodium citrate buffer and 0.1 M EDTA-Tris buffer (Novocastra™ Epitope Retrieval Solutions cat. RE7113, Leica Biosystems). Samples were incubated in 0.9% H_2_O_2_ for 5 min followed by 1 h blocking in 1% BSA in PBS [23]. Slides were incubated for 1 h at room temperature with anti-KI67 (1:100, Clone SP6 CRM325, BIOCARE Medical), washed and incubated in MACH1 Universal HRP-polymer (Cat. MRH538G BIOCARE Medical) for 1 h at room temperature. Then, samples were developed with Betazoid DAB Chromogen Kit (Cat. BDB2004H, BIOCARE Medical), counter-stained with hematoxylin and mounted with synthetic resin solution (Entallan .107960 Merck).

### TUNEL assay

Apoptosis was analyzed in tissue samples using the TUNEL (terminal deoxynucleotidyl transferase dUTP nick end labeling) in situ cell death detection kit (ROCHE) according to the manufacturer’s instructions. Briefly, paraffin sections of colon tumor samples were deparaffinized in xylene, rehydrated in a series of graded alcohols, fixed in 4% paraformaldehyde (PFA, Sigma-Aldrich) for 30 min and digested with 20 μg/mL proteinase K for 15 min at room temperature. Tissue samples were stained using the TUNEL reaction mixture. Nuclei were stained with DAPI and samples were analyzed by confocal microscopy using an Olympus FV300 microscope.

### Statistical analysis

Statistical comparisons were performed with one-way analysis of variance (ANOVA) followed by Dunnett’s multiple comparisons test using GraphPad Prism 5.0 software. The data are shown as mean ± SEM. A *p* value of < 0.05 was considered statistically significant.

## Results

### The small molecule C19 binds and stabilizes the KRAS4b-PDEδ complex

**Fig. 1:**
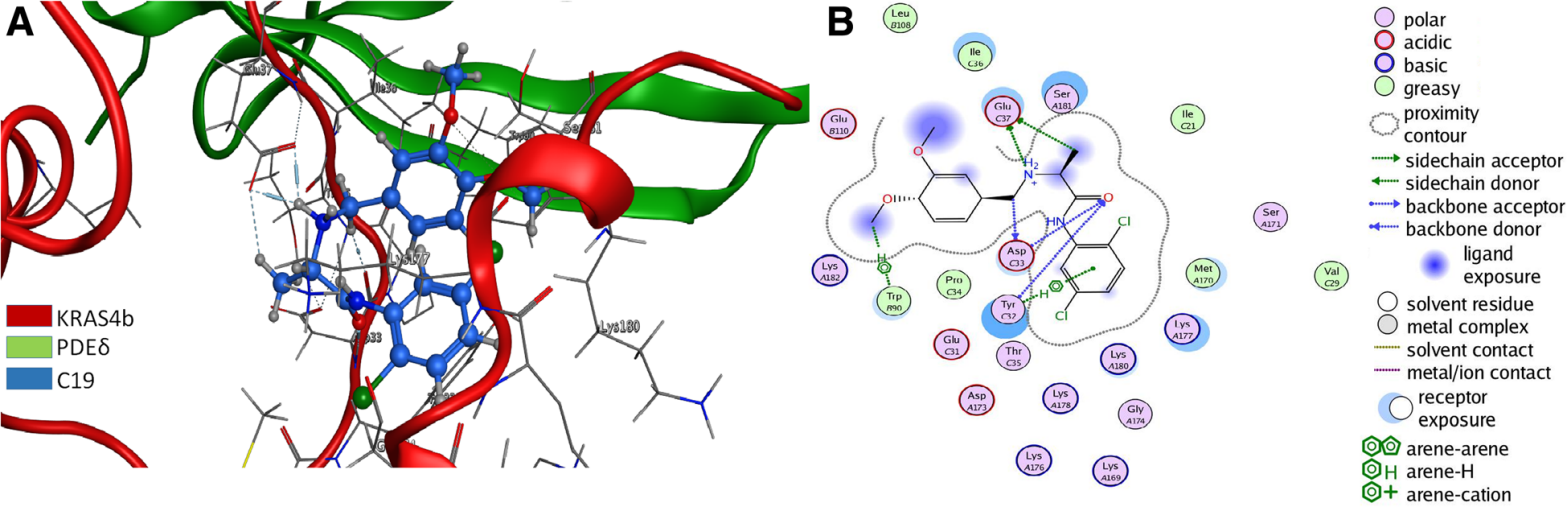
C19 binds and stabilizes the KRas4B-PDEδ complex. **a** Most populated conformation during the equilibrium simulation time. Structural superposition of crystalized KRAS4b (red) in complex with PDEδ (green) interacting with C19 (blue). **b** Interactions of the KRAS4b-PDEδ complex with C19. Interacting amino acids are highlighted

One of the best scoring compounds identified by docking with ENAMINE compound-library [9] termed C19, is a small organic molecule with good pharmacological properties such as low molecular weight of 383.3 g/mol, two hydrogen bond donors and two Cl atoms capable of making halogen interactions and Cl-π interactions with aromatic residues. It also has four hydrogen bond acceptors and a LogP of 3.363 (Additional file 1: Table S1). The docking procedure was intended to find compounds able to simultaneously bind to KRAS2b and PDEδ, thus acting as molecular staple of this complex. To analyze all the potential interactions with the *KRAS4b-PDEδ* complex, we performed further docking studies of the whole interface region of the KRAS4b-PDE**δ** complex with PDB ID code 5TAR [24], where the amino acids of both proteins are in close proximity (Fig. 1a). The docking results were analyzed by the binding score, the frequency of obtaining similar poses, and the kind of non-bonded interactions. Visual inspection of the docked pose of C19 (blue) showed simultaneous interactions with KRAS4b (green) and PDE**δ** (red) (Fig. 1a and Additional file 2: Table S2). In the most populated conformation obtained from the equilibrated simulation time (50 ns), the C19 methylamine has five interactions with the Glu 37 of KRAS4b via electrostatic and hydrogen bond interactions with a distance between 3.19 and 2.81 Å. C19 also has hydrogen bond and π-π interactions with Tyr 32 and hydrogen bond interactions with Asp 33 of KRAS4b. Moreover, C19 forms hydrogen and π-π interactions with Trp 90 of PDE6δ with a distance of 4.71 Å (Fig. 1b, Additional file 2: Table S2). In the first conformation of the DM simulation, C19 forms six interactions with PDEδ and five with KRAS4b (Additional file 3: Figure S1A). In addition, in the first conformation of the DM simulation, C19 maintains strong interactions with Glu 106 of PDEδ via hydrogen bond interactions and with Ser 15 of KRAS4b (Additional file 3: Figure S1B). Based on this structural analysis, subsequent clustering analysis and free binding energy calculations were performed for the KRAS4b-PDEδ- farnesyl group (which is a physiological posttranslational modification of KRAS4b) and KRAS4b-PDEδ- farnesyl group-C19 excluding the first 50 ns from the 100-ns-long MD simulation. Indeed, *ΔG*_*bind*_ values were energetically more favorable between KRAS4b-PDE**δ** and C19 than with only a farnesyl group suggesting that C19 has the potential to stabilize the KRAS4b-PDE**δ** complex thus preventing its dissociation (Additional file 4: Table S3).

### C19 has specific cytotoxic activity in KRAS4b-dependent colon cancer cells

**Fig. 2:**
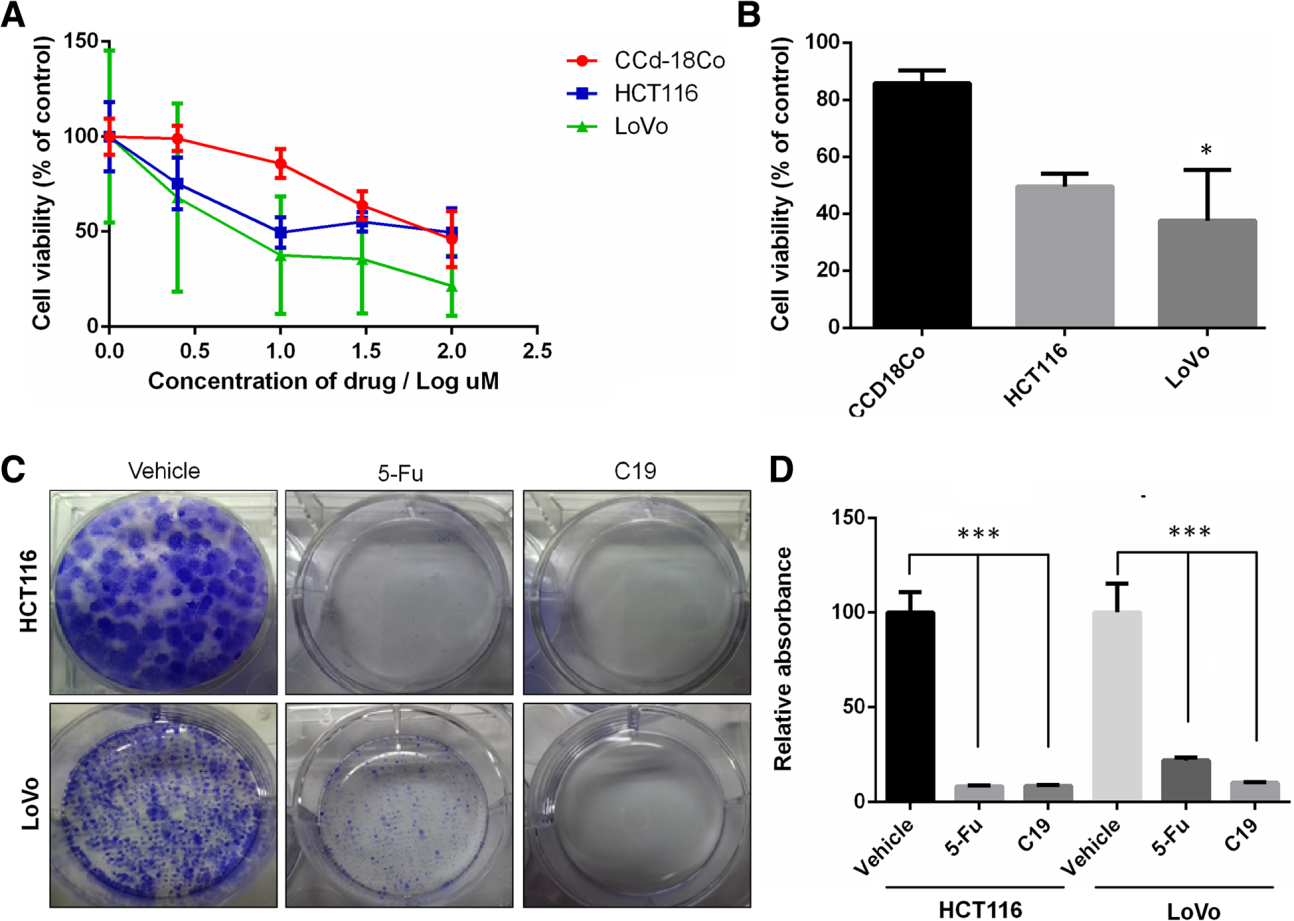
C19 inhibits the growth of human colorectal cancer cell lines. **a** The colorectal cancer cell lines HCT116, LoVo and the normal colon cell line CCD-18Co were treated with the indicated concentrations of C19 for 72 h. IC_50_ values of HCT116, LoVo and CCD-18Co cells were 103.03, 22.7 and 60.8 μM, respectively. **b** Treatment with C19 at a concentration of 10 μM significantly reduces the viability of the LoVo cell line. *p < 0.01 (**c**) Cells were treated with vehicle (DMSO), the IC_50_ of C19 and IC_85_ of 5-Fu in a clonogenic assay and stained with crystal violet. A representative plate of three independent experiments is shown. **d** Quantification of crystal violet staining from cells depicted in (**c**). *n* = 3; *** *p* < 0.0001

Given the improved affinity of the KRAS4b-PDEδ complex through binding of C19, and the importance of this complex in certain types of colorectal cancer, we wanted to know whether C19 affects the viability of KRAS-dependent colorectal cancer cells. We used the KRAS-dependent metastatic colorectal cancer cell line LoVo, the KRAS-independent colon carcinoma cell line HCT116 and the normal colon cell line CCD-18Co as control [25]. Cell viability was determined by measuring ATP concentrations in the cell lines after 3 days of treatment with C19 at different concentrations (2.5, 10, 30 and 100 μM). We found that the KRAS-dependent LoVo cell line was more sensitive to treatment in a dose-dependent manner with an IC_50_ of 22.7 μM; whereas the HCT116 cell line and the normal cell line CCD-18Co had much higher IC_50_ values of 103.03 and 60.8 μM, respectively (Fig. 2a). Of note, at a concentration of 10 μM, C19 showed significantly higher cytotoxic effects in LoVo cells than in CCD-18Co cells, while we did not observe significant differences between HCT116 and CCD-18Co cells (Fig. 2b). Given the known function of KRAS4b in cell proliferation, we next determined whether C19 regulates cell growth using a colony-forming assay to evaluate the capacity of a single cancer cell to expand after three doses of either C19 or 5-Fluorouracil (5-Fu, a drug currently used in colon cancer treatment). Both drugs significantly reduced cell proliferation and colony formation (Fig. 2c). Importantly, C19 reduced the formation of colonies to a greater extent compared to 5-Fu treatment only in the KRAS-dependent LoVo cells (Fig. 2d). These results suggest that C19 has strong specific cytotoxic activity in colon cancer cells dependent on oncogenic KRAS4b activity.

### C19 induces apoptosis

**Fig. 3:**
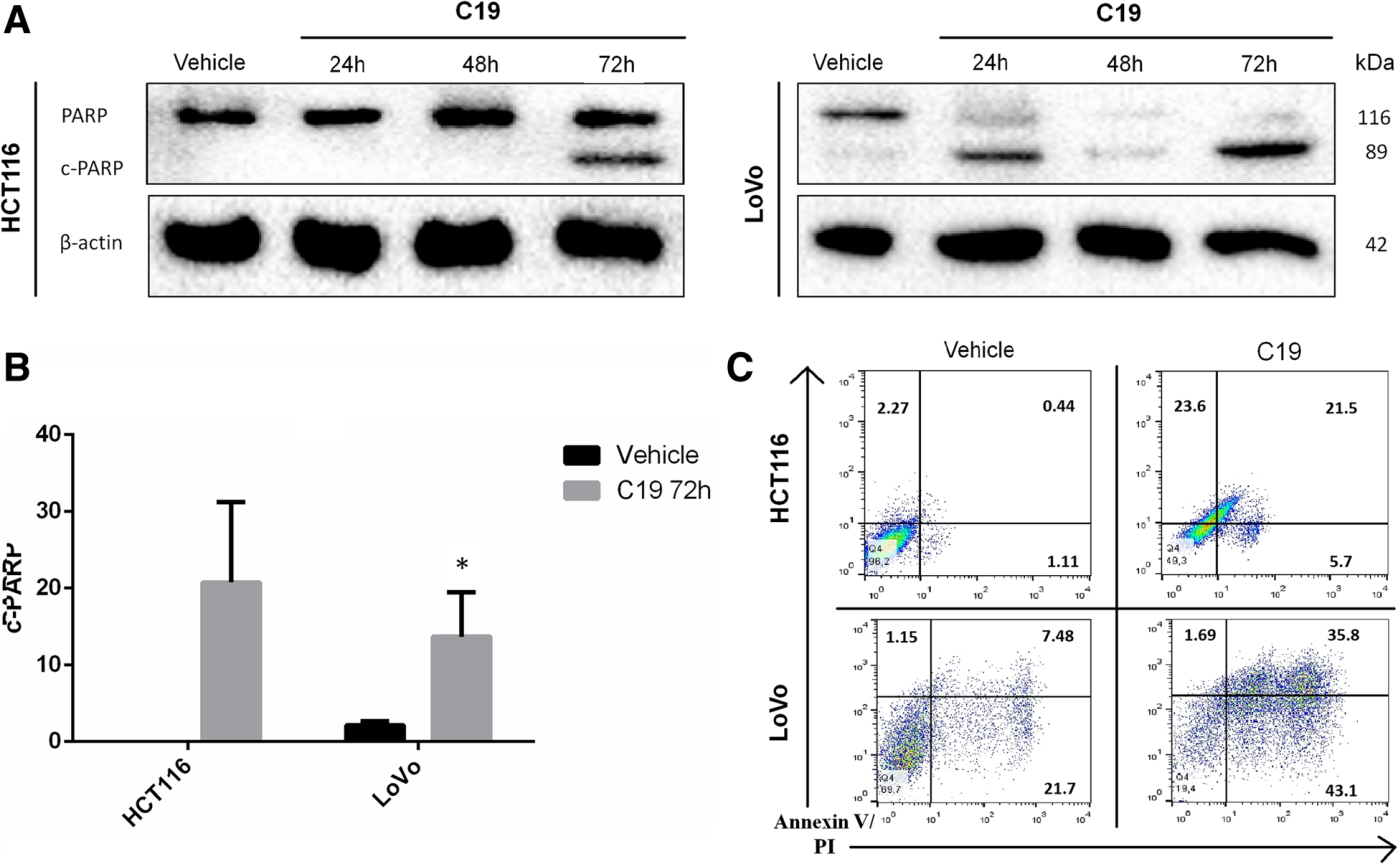
C19 induces apoptosis in colorectal cancer cells. **a** Cells were treated with the IC_50_ of C19 for 24, 48 and 72 h and cell lysates were analyzed for cleaved PARP. β-actin served as loading control. Representative blots of three independent experiments are shown. **b** Densitometric analysis of c-PARP normalized to total PARP levels in HCT116 and LoVo cells treated with C19 for 72 h. Error bars represent SEM. *p < 0.01 (**c**) Annexin V and PI analysis of HCT116 and LoVo cells treated with the IC_50_ concentration of C19 or vehicle alone for 72 h. C19 treatment strongly increased the percentages of LoVo cancer cells in early (Annexin V+ PI-) and late apoptosis (Annexin V+ PI+)

The observed cytotoxic effect prompted us to investigate how C19 inhibits viability and proliferation of KRAS-dependent colon cancer cells. To this end, we performed western blots to detect if the poly-(ADP-ribose)-polymerase (PARP) protein is cleaved (c-PARP) after 24, 48 and 72 h of treatment with C19 in both cancer cell lines. C19 promotes the cleavage of PARP already after 24 h in the LoVo cell line (Fig. 3a right). Interestingly, only after 72 h of treatment, c-PARP can be observed in the HCT116 cell line (Fig. 3a left). C19 only promotes a low percentage of apoptosis in normal CCD-18Co cells and does not cause necrosis in these cells (Additional file 5: Figure S2A). The increase in the cleavage of PARP at 72 h post-treatment with C19 is significant in both cancer cell lines compared to the vehicle (DMSO) (Fig. 3b). Therefore, we analyzed KRAS-dependent LoVo cells and KRAS-independent HCT116 cells by flow cytometry using Annexin V and propidium iodide (PI) after C19 treatment using the respective IC_50_ concentrations or DMSO as a vehicle (Fig. 3c). Vehicle treatment did not significantly affect cell death compared to untreated cells (data not shown). By contrast, treatment with C19 caused an increase in late apoptosis (annexin V^+^ PI^+^) of approximately 23% in HCT116 cells and 28.3% in LoVo cells. However, in LoVo cells there is also an increase of necrosis (annexin V^−^ PI^+^) of approximately 20% compared to HCT116 with only a 5% increase. Overall, the results suggest that treatment with C19 causes cell death by apoptosis in both cancer cell lines.

### C19 treatment reduces KRAS4b-dependent signaling

**Fig. 4:**
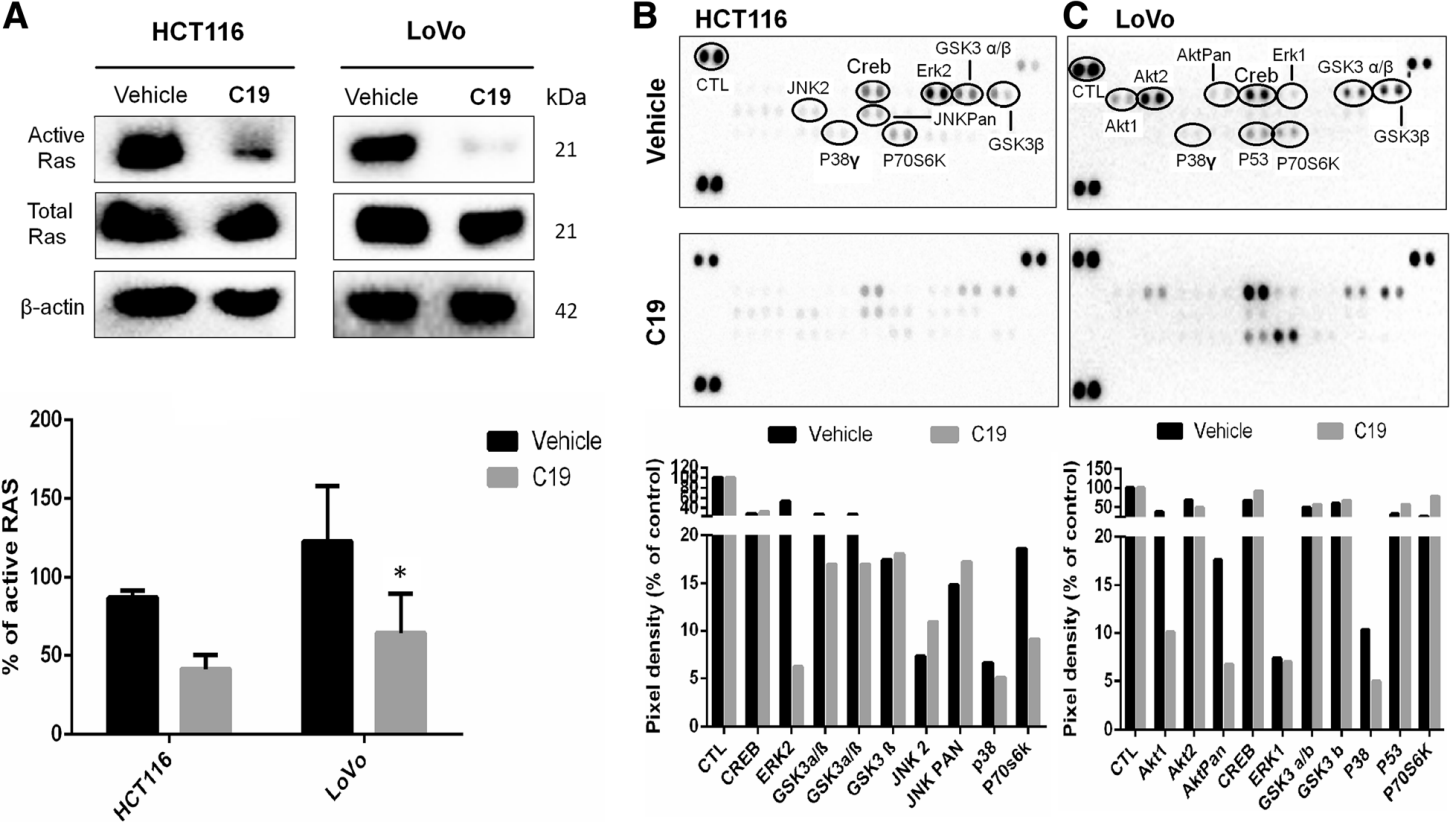
The effect of C19 treatment on RAS activation and MAPK phosphorylation. **a** Representative western blot image of HCT116 and LoVo cell lines treated with vehicle and C19 (*n* = 3). Total protein extracts were challenged with RAF-RBD beads. The membrane was probed with antibody to pan-RAS, provided with the kit, to confirm pull down of active RAS. Total extracts were immunoblotted with the antibodies as indicated and densitometric analysis in arbitrary units. *p < 0.01 (lower panel). **b, c** Phosphoproteome profiling of HCT116 (**b**) and LoVo cells (**c**) treated with vehicle (upper panel) and IC_50_ concentration of C19 (lower panel) for 24 h. Total cell lysates from colon cancer cell lines after treatment were incubated on membranes of the phosphoproteomic human Phospho-MAPK kit as described in methods. Quantification of the pixel intensities of the signals in duplicates each from one membrane is shown. Data are depicted as percentage of control (set to 100%).

To test whether C19 can prevent the activation of RAS in colorectal cancer cells, we performed RAS-GTP pull down assays (Fig. 4a, upper panel). Effectively, C19 significantly inhibits activation of RAS by more than 50% in both HCT116 and LoVo cells 24 h after treatment (Figs. 4a, lower panel). As KRAS4b is known to induce activation of mitogen-activated protein kinases (MAPK), we asked whether treatment with C19 would inhibit signaling downstream of KRAS4b. We employed a commercial membrane containing antibodies that recognize 28 different phosphorylated proteins of the MAPK family (Fig. 4b and c). After 24 h of treatment with C19, KRAS-independent HCT116 cells showed slight increases of activation of JNK2 and JNKpan, which are usually activated in response to cell stress [26] suggesting that JNK signaling may have a compensatory effect in this cell line against C19 treatment (Fig. 4b, membrane with duplicate spots of each enzyme; and quantification of the membrane signals in the graph below). By contrast, C19 treatment decreased the phosphorylation by 50% of kinases involved in de novo protein synthesis such as p70s6K, and significantly decreased Erk2 activation by 88.5% (Fig. 4b). Interestingly, C19 treatment in KRAS-dependent LoVo cells showed significant inhibition of the phosphorylation of Akt1 (74%) and Aktpan (61.7%), and a small decrease in Erk1 (4.1%) phosphorylation. In addition, C19 affects the phosphorylation of the amino acids T421/S424 of p70s6K (Fig. 4c, and quantification of the membrane signals in the graph below). Interestingly, we identified that after treatment with C19 in both cell lines, the activation of p38 decreased by 24–50%. By contrast, in the normal colon cell line CCD-18Co, C19 rather increased the activation of kinases involved in the KRAS4b pathway such as Erk1, Akt1 and Akt2 (Additional file 5: Figure S2B and S2C). These data suggest that C19 decreases activation of many KRAS4b-dependent pathways specifically in colorectal cancer cell lines.

### C19 treatment inhibits tumor growth of xenotransplanted LoVo cells

**Fig. 5:**
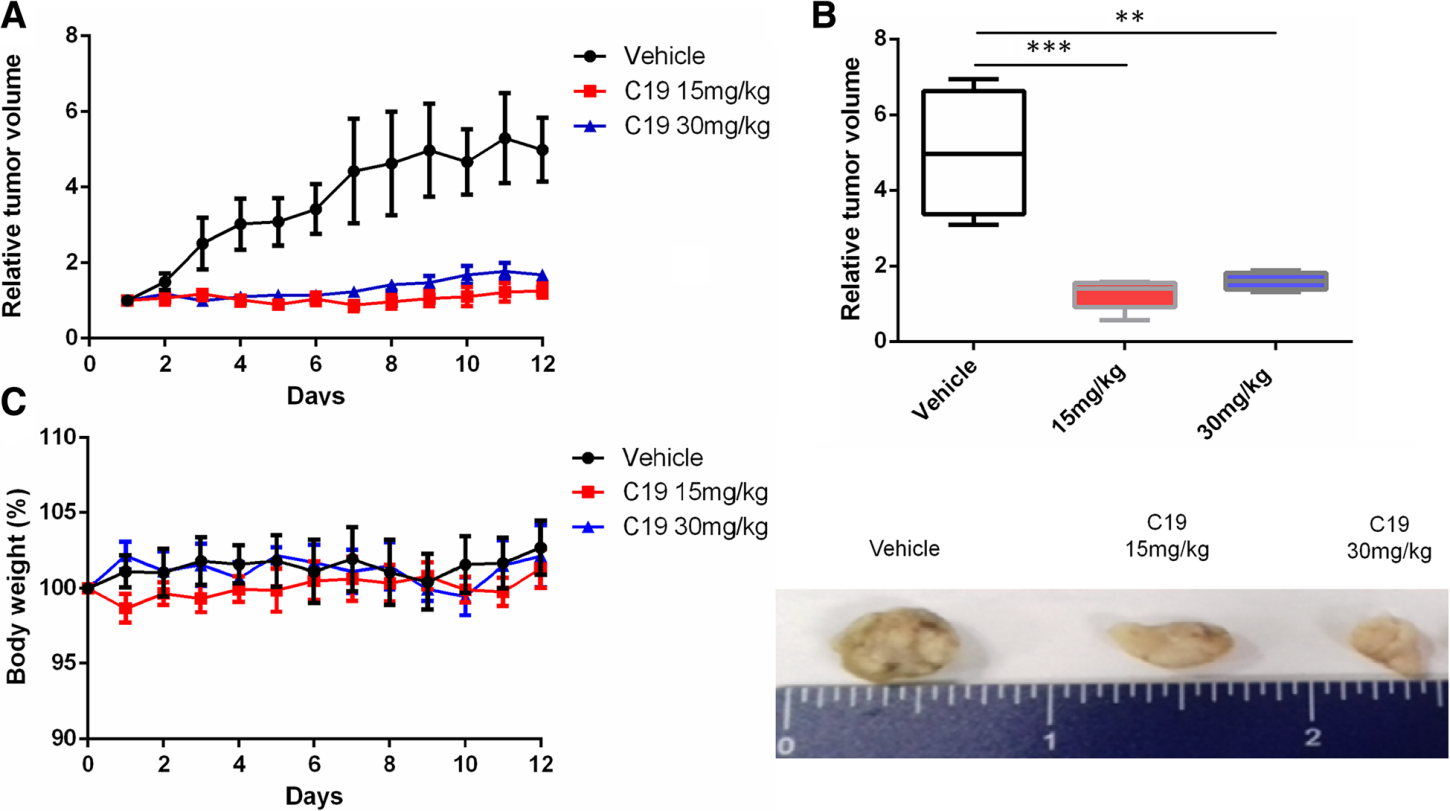
C19 treatment reduces tumor size in vivo. **a** Intraperitoneal (i.p.) C19 treatment at 15 mg/kg and 30 mg/kg (*n* = 6) daily during 12 days reduces the tumor size in a murine colon cancer xenograft model after injection of LoVo cells. The mean percent changes in tumor volume relative to initial tumor volume are shown. Error bars represent SEM. **b** Tumor volume distribution after 12 days of LoVo engraftment with vehicle or C19 treatment. *n* = 6 **p < 0.001; ***p < 0.0001 (upper panel). General observation of tumors at the end of the experiment (lower panel) (**c**) Body weight was measured daily during treatment. C19 at both concentrations tested did not lower body weight compared to vehicle treatment. Data represent means ± SD of at least three independent experiments

**Fig. 6:**
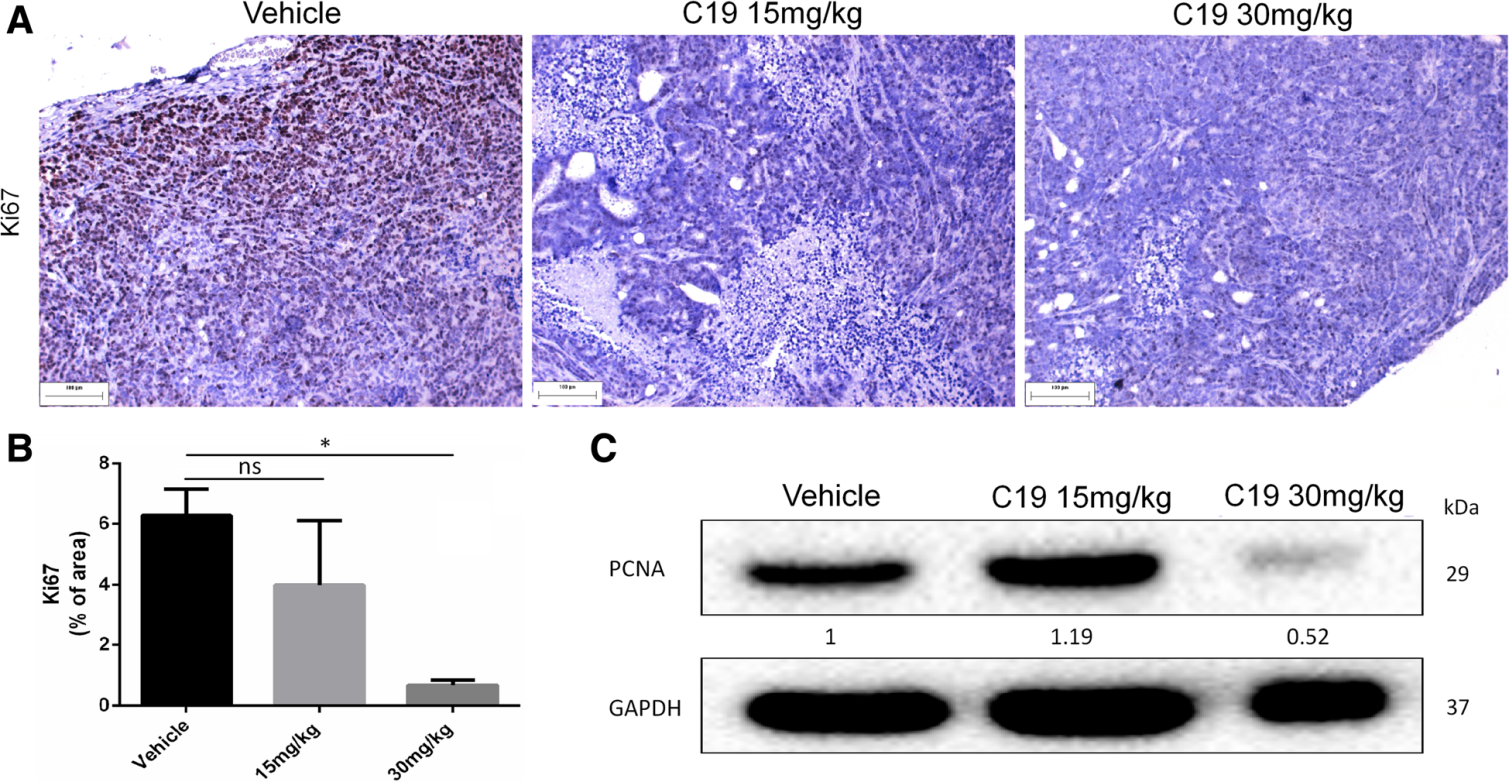
C19 treatment inhibits cell proliferation in vivo. **a** Tumor tissues from LoVo xenografts were evaluated by immunohistochemistry for Ki67 (a marker of proliferation). **b** The graph shows signal intensities of positive cells calculated using Image Pro Plus software. *n* = 3 *p < 0.05. **c** Tumor lysates were analyzed by western blot for expression levels of proliferating cell nuclear antigen (PCNA). Densitometric analysis was performed with Image Lab 4.0

Given the specific cytotoxic effect of C19 in KRAS-dependent LoVo cells in vitro, we next tested whether C19 exhibits antitumor activity in a colorectal cancer xenograft model using LoVo cells. The xenograft tumor was established by subcutaneous injection of 5 × 10^6^ LoVo cells in Nu/Nu mice. The mice were then treated with vehicle, 15 or 30 mg/kg C19 intraperitoneally once daily for a total experimental period of 12 days. Over the entire period of C19 administration, we observed a significant decrease in tumor growth with both concentrations of C19 in comparison with the administration of vehicle (Fig. 5b upper panel). Extracted tumors after 12 days showed that C19 treatment strongly reduced tumor size (Fig. 5b lower panel). We did not observe any significant changes in body weight of the differently treated mice over the entire course of the experiment (Fig. 5c). Immunohistochemical analyses of tumor tissues revealed that C19 significantly decreased cell proliferation as measured by Ki-67 staining (Fig. 6a). C19 at a concentration of 15 mg/kg decreased Ki67 staining by 30–40% and by more than 75% at a concentration of 30 mg/kg (Fig. 6b). To corroborate the effect of C19 on the proliferation of tumor cells in vivo, we did western blots to identify the expression of proliferating cell nuclear antigen (PCNA). Indeed, at 30 mg/kg of C19, PCNA decreased by 48% (Fig. 6c). Moreover, and in agreement with our in vitro data, C19 treatment increased apoptosis as determined by TUNEL staining. Tumors treated with compound C19 at 15 mg/kg present TUNEL signals only at the periphery (Additional file 6: Figure S3A). By contrast, TUNEL-positive cells were significantly elevated in tumors treated with C19 at 30 mg/kg (Additional file 6: Figure S3A and B).

### C19 prevents phosphorylation of Akt and Erk in vitro and in vivo

**Fig. 7:**
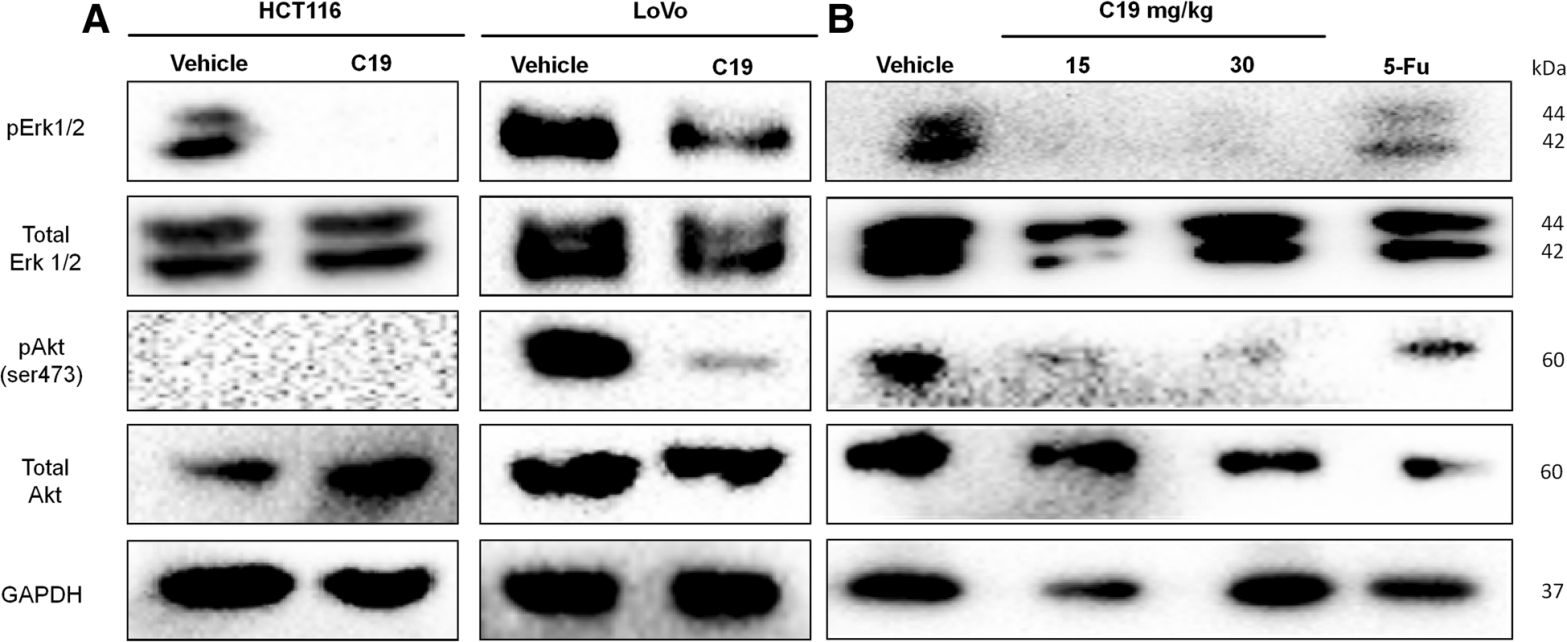
C19 prevents phosphorylation of Erk and Akt in vitro and in vivo. **a** Cell lysates and (**b**) tumor lysates were analyzed by Western blot for phosphorylation levels of ERK (p-Erk) and Akt (P-Akt). Total Erk, total Akt and β-actin served as loading control. Representative blots of three independent experiments are shown

Finally, we analyzed whether C19 prevents the phosphorylation of Erk1/2 and Akt in the LoVo-derived xenograft tumors similar to what we observed in vitro (Fig. 4). Akt is a known downstream target of active KRAS4b that has been directly correlated with oncogenic KRAS4b activity [27]. Figure 7a confirms the data of the phospho-MAPK assay by Western blot in which we also saw that treatment with the IC_50_ of C19 for 24 h significantly inhibited the phosphorylation of Erk1/2 in both colorectal cancer cell lines. Importantly, Akt was only active under basal conditions in LoVo cells and was significantly inhibited by C19. Analysis of total protein extracts from xenograft tumors showed similar levels of total Erk1/2 and Akt in all samples (Fig. 7b). Importantly, C19 treatment almost completely abolished Erk1/2 and Akt phosphorylation in vivo, and this effect was more pronounced in comparison to 5-fluorouracil (5FU) treatment.

## Discussion

Metastatic colorectal cancer remains one of the most lethal cancers, and this pathology has few treatment options when mutated KRAS4b protein is found. In the present study, we developed a new strategy to inhibit selectively the activity of mutated KRAS4b. Using bioinformatics studies such as docking and MD simulations, we detected that C19 binds and stabilizes the molecular complex KRAS4b-PDEδ by binding to the heterodimer interface. In recent years, various researchers rather tried to selectively inhibit one or the other protein of the KRAS4b-PDEδ complex. Small molecules that irreversibly bind to mutated oncogenic KRAS have been developed [28]. Binding of these inhibitors interrupted both switches (I and II) of KRAS altering the preference of native nucleotides to favor GDP over GTP. One of the disadvantages is that these small molecules only work with the KRAS G12C mutation. Moreover, this mutation is not the prevalent in colorectal cancer and the authors did not perform anti-tumorigenicity tests. We selected C19 due to its binding properties on the KRAS4b-PDEδ complex suggesting that it does not interfere with the switch of KRAS4b but that it rather stabilizes the complex thus increasing the free binding energy of the KRAS4b-PDEδ-Far-C19 system compared to the KRAS4b-PDEδ-Far system. Other strategies were based on reaching the hydrophobic pocket of PDEδ to inhibit binding of KRAS4b and thus avoid its transport to the plasma membrane. For example, deltarasin can bind to the hydrophobic pocket of PDEδ and selectively inhibits the viability of KRAS-dependent pancreatic ductal cancer cells [29]. Our bioinformatic strategy used for drug design has been explored before to obtain potential drugs with higher efficiency to disrupt protein-protein interactions (https://www.ncbi.nlm.nih.gov/pubmed/28482793). Here, we provide experimental evidence that C19 selectively inhibits the viability of KRAS-dependent colorectal cancer cells.

C19 inhibits the viability and proliferation of colorectal cancer cells by induction of apoptosis. C19 significantly increased cleaved PARP in the KRAS-dependent cell line after 24 h of treatment. In colorectal cancer cells with mutated KRAS, treatment with cetuximab (anti-EGFR mAbs) or regorafenib (oral multikinase inhibitor) alone does not produce more than 22% of apoptosis after 24 h of treatment [30]. From a therapeutic standpoint, suppression of apoptosis is probably one of the most important consequences of KRAS mutations [31]. Here, C19 has superior effects on the inhibition of viability, proliferative capacity and induction of apoptosis in KRAS-dependent colorectal cancer cells.

**Fig. 8:**
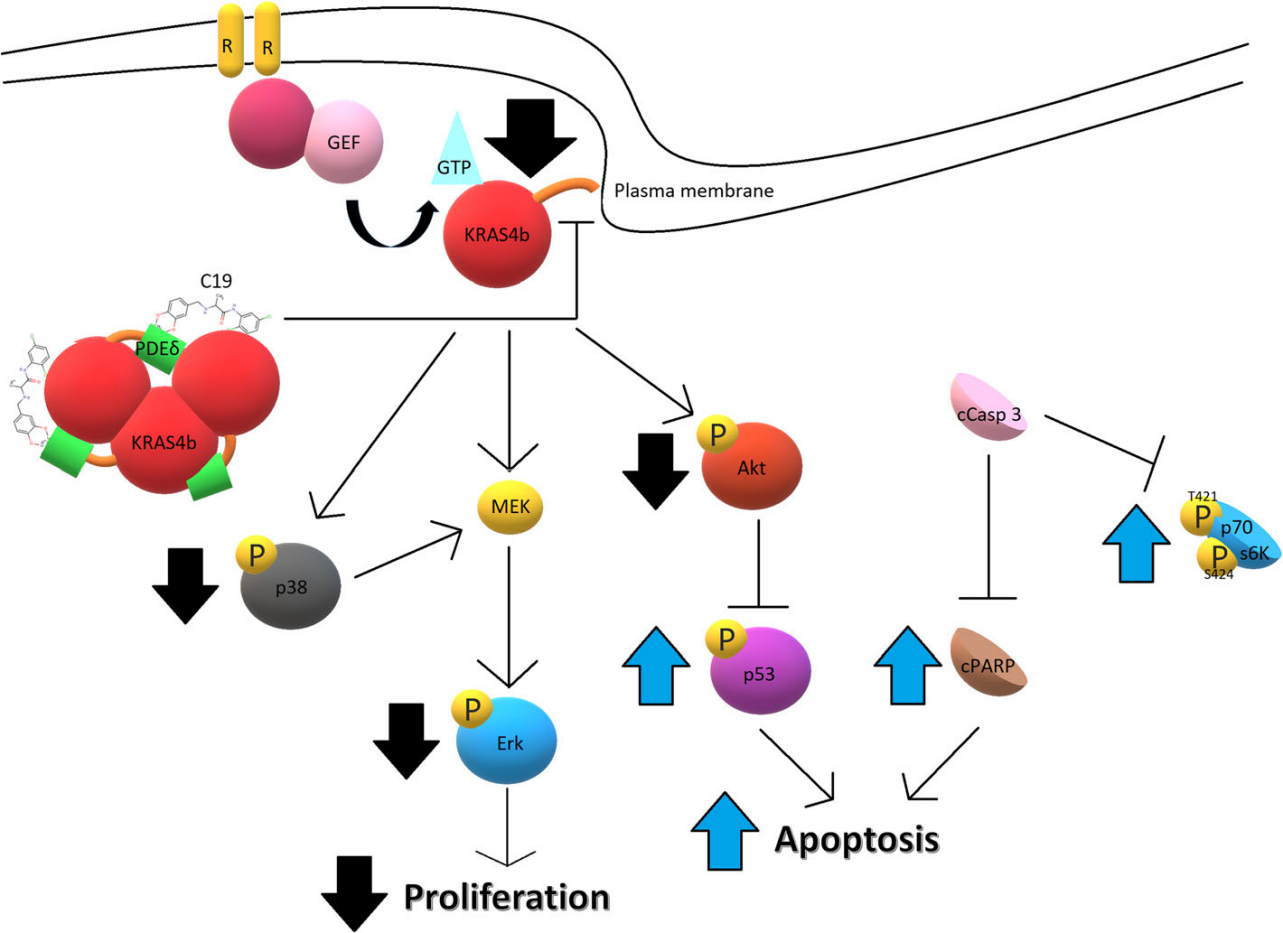
Schematic representation of the effect of C19 on the activation of KRAS4b and oncogenic KRAS4b signaling. C19 significantly reduces cellular proliferation by inhibition of the Erk and p38 pathway, and induces apoptosis by inhibiting Akt activation

Inhibitors of PI3K/Akt signaling have been suggested as potential therapeutic agents in colorectal cancer. Our data show that C19 decreased the activation of Akt1, Akt2 and AktPan and increased the activation of p53, which is important since the phosphoinositide 3-kinase (PI3K)-Akt signaling pathway plays a prominent role in tumorigenesis, progression of colorectal cancer and anti-apoptosis pathway [32]. These results correlate with the increase in the concentration of c-PARP after treatment with C19 (Fig. 8). Indeed, activated KRAS can inhibit the apoptotic signaling cascade through its effector PI3K, which in turn activates Akt (a potent pro-survival kinase) that inhibits apoptosis via several mechanisms including phosphorylation and subsequent inactivation of the pro-apoptotic Bcl-2 family protein BAD, and the inhibitory phosphorylation of caspase-9 [31].

Interestingly, after treatment with C19 in the LoVo cell line, p70S6K showed increased phosphorylation signal (Fig. 4c). This is of relevance because lung cancer cells also had an increase in the signal of the phosphorylated 45 kDa fragment of p70S6K in response to apoptosis caused by cisplatin [33]. With respect to our data, this suggests that the increase in p70S6K signal may be derived from the cleaved protein, in response to apoptosis caused by C19 (Fig. 8). In addition, in the LoVo cell line, p38 phosphorylation decreased by more than 50% (Fig. 4c). In colorectal cancer, activation of p38 by mutated KRAS has been implicated in the maintenance of cell proliferation due to inhibition of MEK [34], suggesting that C19 inhibits the activation of KRAS4b thus leading to the inhibition of activation of its multiple effectors involved in oncogenic signaling such as proliferation and anti-apoptotic pathways (Fig. 8).

Furthermore, in the in vivo xenograft model, C19 at concentration of 15 and 30 mg/kg caused a significant decrease in the phosphorylation of Erk1/2 and Akt, thus confirming inhibition of KRAS4b signaling in vivo. Inhibitors that affect signaling downstream of KRAS4b such as the RAF inhibitor LY3009120 significantly inhibited tumor growth in murine models of skin and colon cancer, with a dose that inhibited 50% of Erk phosphorylation (ED_50_) of 4.36 mg/kg. However, concentrations of 12.5 to 25 mg/kg did not completely reduce phosphorylation of Erk [35]. Therefore, it would be important to perform tests at lower concentrations of C19 to find a minimum dose of both inhibition of tumor growth and inhibition of the KRAS4b pathway.

C19 significantly decreased tumor growth and much more efficient than the currently used chemotherapeutic agent 5-Fu, which also showed significant toxicity in mice [36]. 5-Fu is effective against some types of intestinal cancers such as colorectal cancer where it is primarily associated with tissue damaging and regenerating effects in the gastrointestinal mucosa [37]. FOLFOX4 is a combination chemotherapy regimen involving administration of oxaliplatin in combination with 5-FU and leucovorin; this regimen is reportedly active in patients who have been previously treated with 5-FU alone or in combination with leucovorin. However, the FOLFOX4 regimen requires at least three days of hospitalization because of the 48 h continuous intravenous infusion and is unsatisfactory from the perspective of quality of life [38]. In our study, 5-Fu caused rapid weight loss in mice (data not shown). By contrast, C19 administered daily did not cause measurable adverse effects in mice. In addition, we observed decreased cell proliferation in xenograft tumors as measured by Ki67corroborating the observed decrease in cell proliferation in vitro*.* In a small study of chemosensivity prediction, Ki67 was used as a predictor of the treatment response and it was effectively demonstrated that treatment-sensitive tumors showed a significant decrease in Ki67 expression compared to resistant tumors [39]. In this respect, C19 could be potent regulator of the balance between cell proliferation and apoptosis in colorectal cancer cells.

## Conclusions

In conclusion, we identified a new compound that functions as a stabilizer of the KRAS4b-PDEδ complex, and that decreases the proliferation of colorectal cancer cells, and increases apoptosis via decreased activation of oncogenic KRAS4b signaling. It will be important to explore antitumor properties of C19 in patient-derived colon tumor xenograft (PDTX) mouse models. C19 has the potential to be an alternative treatment for colorectal cancer dependent on mutated KRAS4b.

## Additional files

Additional file 1: **Table S1.** Characteristics of the C19 compound. (DOC 27 kb)

Additional file 2: **Table S2.** MD simulation results of the first populated site of the C19 compound in the KRAS4b-PDEδ complex. (DOC 40 kb)

Additional file 3: **Figure S1.** Conformation 1 of the KRAS4b-PDEδ-C19 complex. (A) Conformation 1 of the KRAS4b-PDEδ-C19 complex where it can be observed that compound C19 is extended, interacting with both proteins by forming six bonds with PDEδ and five with KRAS4b (B) Interactions of the protein-ligand complex with C19 in the first conformation of DM simulation. Interacting amino acids are highlighted. (TIF 717 kb)

Additional file 4: **Table S3.** Binding free energy components of protein-ligand complexes (in kcal/mol units). Binding free energies and individual energy terms of bound PL complexes with ORL or EGCG starting from docked conformations (kcal/mol). The polar (*ΔE*_*polar=*_*ΔE*_*ele*_ *+ ΔG*_*ele,sol*_) and non-polar (*ΔE*_*non-polar=*_*ΔE*_*vwd*_ *+ ΔG*_*npol,sol*_) contributions. All the energies are averaged over 500 snapshots at time intervals of 100 ps from the last 50 ns-long MD simulations and are in kcal/mol (± standard error of the mean). (DOC 30 kb)

Additional file 5: **Figure S2.** C19 promotes cell death by apoptosis in a small percentage of cells of the normal colon cell line and increases phosphorylation of Erk and Akt. (A) Annexin V and PI staining of the normal cell line CCD-18Co treated with 22.7 μM of C19 or vehicle alone for 72 h. C19 treatment slightly increased the number of colorectal cancer cells in early (Annexin V+ PI-) and late apoptosis (Annexin V+ PI+). (B) Phosphoproteome profiling of CCD-18Co cells treated with vehicle (upper panel) and 22.7 μM of C19 (lower panel) for 24 h. Total cell lysates from normal colon cell lines after treatment were incubated on membranes of the phosphoproteomic human Phospho-MAPK kit as described in methods. (C) Quantification of the pixel intensities of the signals in duplicate each from one membrane. Data are depicted as percent of positive control (set to 100%). (TIF 319 kb)

Additional file 6: **Figure S3.** TUNEL staining of xenograft tumors. (A) Apoptotic cells appear green and nuclei were stained with DAPI (blue). (B) The relative fluorescence units (RFU) of apoptotic cells (green) were calculated using Image Pro Plus software. *n* = 4 *p < 0.01. Error bars represent SEM. Bar = 50 μm. (TIF 9417 kb)

## Abbreviations

CRC: Colorectal cancer
EGFR: Epithelial growth factor receptor
KRAS4b: Kirsten Rat Sarcoma variant 4b
PDEδ: Phosphodiesterase 6 subunit δ
