# Supplementary material for: The small organic molecule C19 binds and strengthens the KRAS4b-PDEδ complex and inhibits growth of colorectal cancer cells in vitro and in vivo
Source: BMC Cancer. 2018 Nov 1;18:1056. doi: 10.1186/s12885-018-4968-3 (PMC6211466; doi:10.1186/s12885-018-4968-3)
Supplement: Supplementary file 1 — Table S1. Characteristics of the C19 compound. (DOC 27 kb) [file 12885_2018_4968_MOESM1_ESM.doc]

**Table SI. Characteristics of the** C19 compound.

| **Comp.** | **IUPAC name** | **Molecular Weight**  **(g/mol)** | **Formula** | **H-Donors** | **H- Acceptors** | **LogP** | **Rotatable bonds** | **Purity** |
| --- | --- | --- | --- | --- | --- | --- | --- | --- |
| **C19** | (2S)-N-(2,5-diclorofenil)-2-[(3,4-dimetoxifenil)metilamino]  propanamida | 383,3 | C_18_H_20_Cl_2_N_2_O_2_ | 2 | 4 | 3.36 | 7 | 95.36% |
