# Supplementary material for: The small organic molecule C19 binds and strengthens the KRAS4b-PDEδ complex and inhibits growth of colorectal cancer cells in vitro and in vivo
Source: BMC Cancer. 2018 Nov 1;18:1056. doi: 10.1186/s12885-018-4968-3 (PMC6211466; doi:10.1186/s12885-018-4968-3)
Supplement: Supplementary file 2 — Table S2. MD simulation results of the first populated site of the C19 compound in the KRAS4b-PDEδ complex. (DOC 40 kb) [file 12885_2018_4968_MOESM2_ESM.doc]

**Table SII. MD simulation results of the first populated site of the C19 compound in the KRAS4b-PDEδ** complex

| Protein | **Ligand** | **Receptor** | **Interaction** | **Distance**  **(Å)** | **E (Kcal/mol)** |
| --- | --- | --- | --- | --- | --- |
| KRAS4b | **CB 18** | **OE2 GLU 37** | **H-donor** | **3.19** | **-0.6** |
|  | **N 22** | **OE1 GLU 37** | **H-donor** | **2.65** | **-6.4** |
|  | **N 22** | **OE2 GLU 37** | **H-donor** | **2.81** | **-13.9** |
|  | **CAF 25** | **O ASP 33** | **H-donor** | **3.42** | **-1.5** |
|  | **O 15** | **CA TYR 32** | **H-acceptor** | **3.34** | **-0.3** |
|  | **O 15** | **N ASP 33** | **H-acceptor** | **3.00** | **-4.0** |
|  | **N 22** | **OE1 GLU 37** | **ionic** | **2.65** | **-12.4** |
|  | **N 22** | **OE2 GLU 37** | **ionic** | **2.81** | **-10.7** |
| PDEδ | **CAP 37** | **6-ring TRP 90** | **H-pi** | **4.71** | **-0.3** |
| KRAS4b | **6-ring** | **CD2 TYR 32** | **pi-H** | **3.63** | **-0.2** |
