# Supplementary figures and images for: The small organic molecule C19 binds and strengthens the KRAS4b-PDEδ complex and inhibits growth of colorectal cancer cells in vitro and in vivo
Source: BMC Cancer. 2018 Nov 1;18:1056. doi: 10.1186/s12885-018-4968-3 (PMC6211466; doi:10.1186/s12885-018-4968-3)

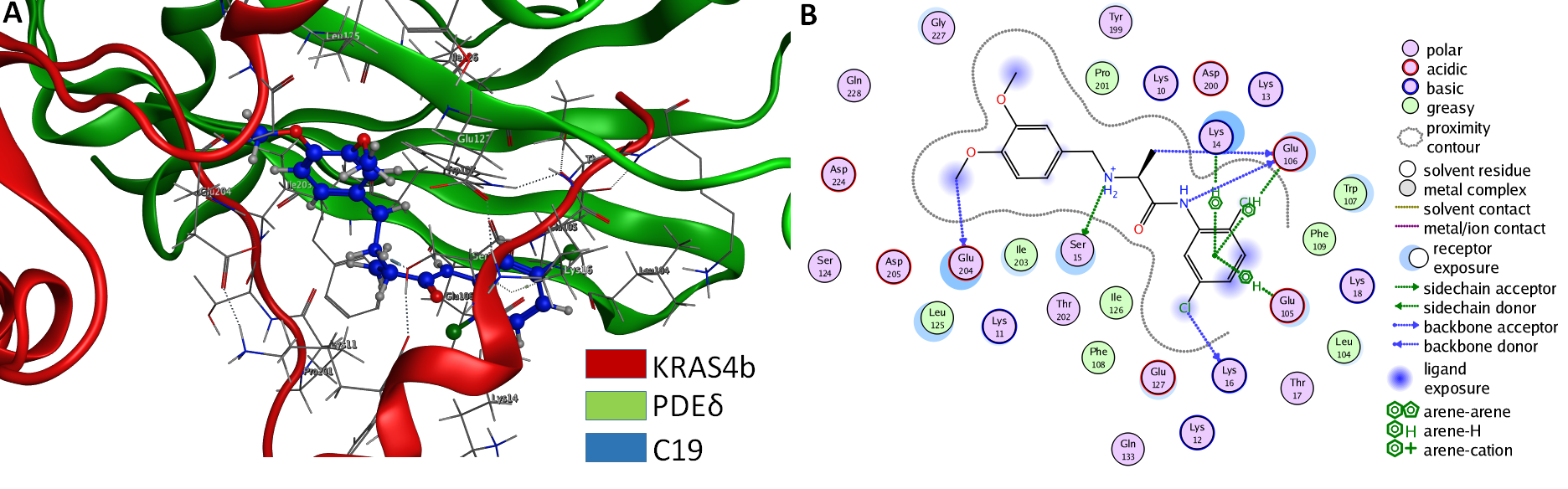

Supplement: Supplementary file 3 — Figure S1. Conformation 1 of the KRAS4b-PDEδ-C19 complex. (A) Conformation 1 of the KRAS4b-PDEδ-C19 complex where it can be observed that compound C19 is extended, interacting with both proteins by forming six bonds with PDEδ and five with KRAS4b (B) Interactions of the protein-ligand complex with C19 in the first conformation of DM simulation. Interacting amino acids are highlighted. (TIF 717 kb) [file 12885_2018_4968_MOESM3_ESM.tif]

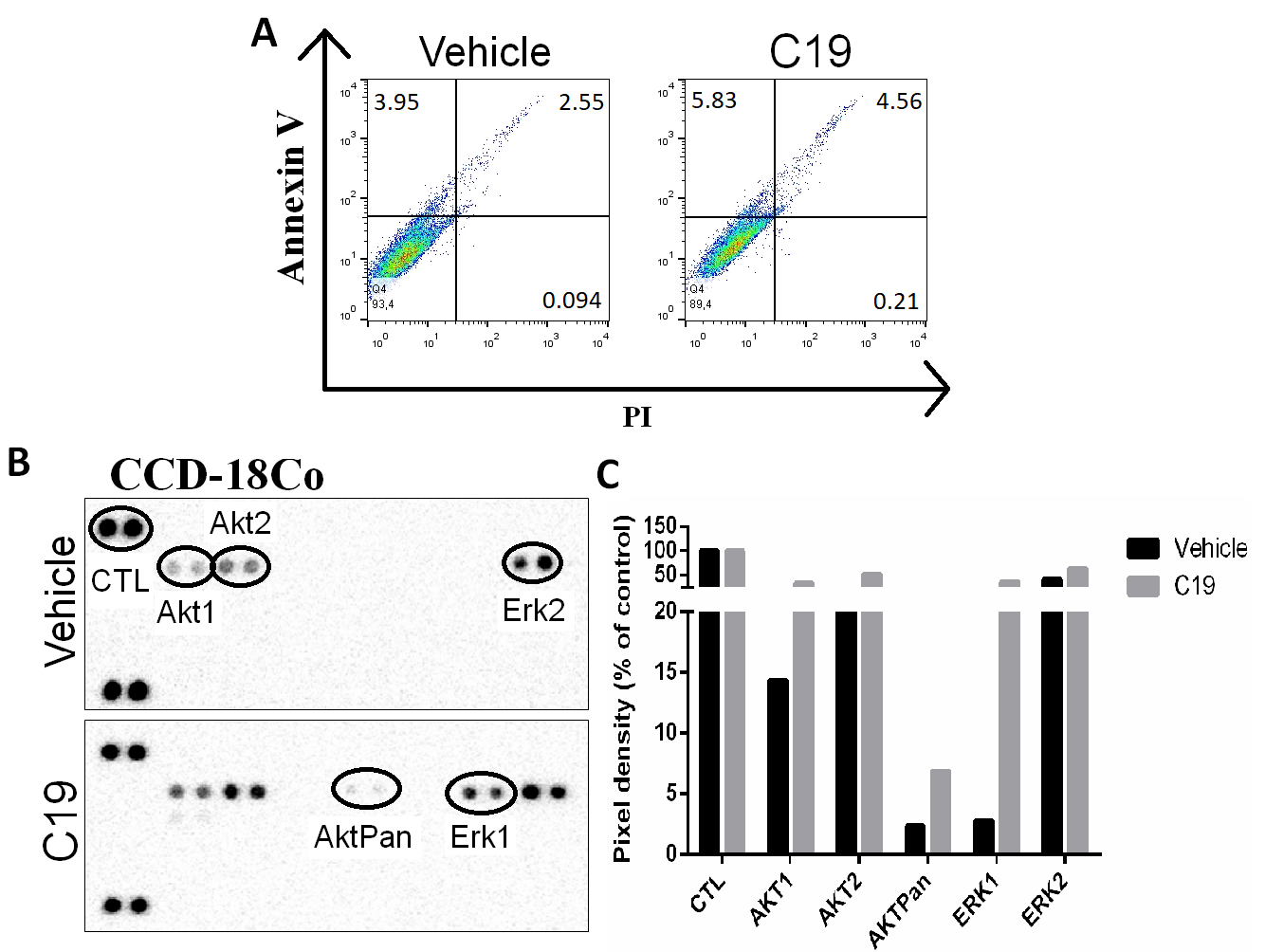

Supplement: Supplementary file 5 — Figure S2. C19 promotes cell death by apoptosis in a small percentage of cells of the normal colon cell line and increases phosphorylation of Erk and Akt. (A) Annexin V and PI staining of the normal cell line CCD-18Co treated with 22.7 μM of C19 or vehicle alone for 72 h. C19 treatment slightly increased the number of colorectal cancer cells in early (Annexin V+ PI-) and late apoptosis (Annexin V+ PI+). (B) Phosphoproteome profiling of CCD-18Co cells treated with vehicle (upper panel) and 22.7 μM of C19 (lower panel) for 24 h. Total cell lysates from normal colon cell lines after treatment were incubated on membranes of the phosphoproteomic human Phospho-MAPK kit as described in methods. (C) Quantification of the pixel intensities of the signals in duplicate each from one membrane. Data are depicted as percent of positive control (set to 100%). (TIF 319 kb) [file 12885_2018_4968_MOESM5_ESM.tif]

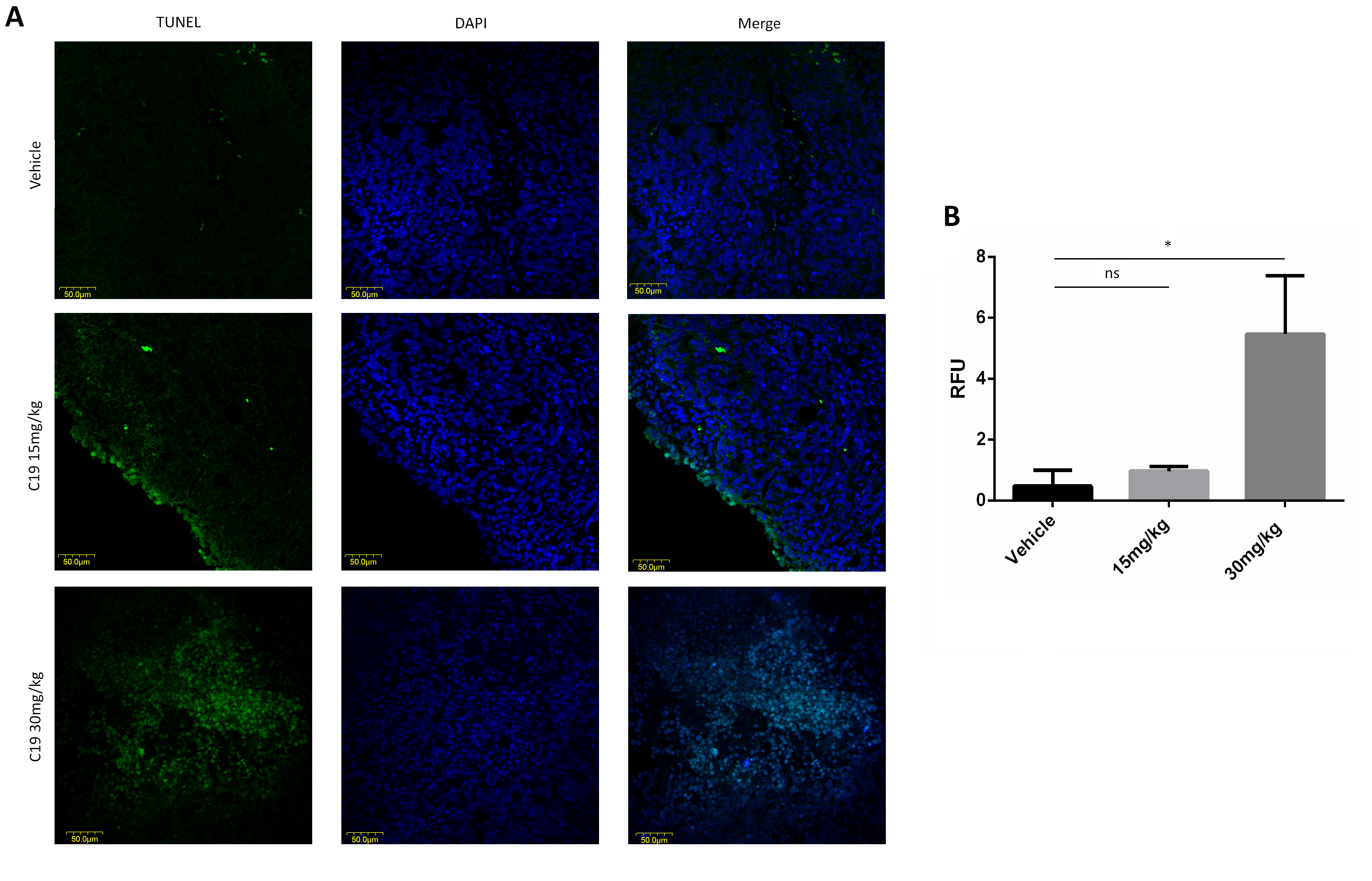

Supplement: Supplementary file 6 — Figure S3. TUNEL staining of xenograft tumors. (A) Apoptotic cells appear green and nuclei were stained with DAPI (blue). (B) The relative fluorescence units (RFU) of apoptotic cells (green) were calculated using Image Pro Plus software. n = 4 *p < 0.01. Error bars represent SEM. Bar = 50 μm. (TIF 9417 kb) [file 12885_2018_4968_MOESM6_ESM.tif]
