# Supplementary material for: The small organic molecule C19 binds and strengthens the KRAS4b-PDEδ complex and inhibits growth of colorectal cancer cells in vitro and in vivo
Source: BMC Cancer. 2018 Nov 1;18:1056. doi: 10.1186/s12885-018-4968-3 (PMC6211466; doi:10.1186/s12885-018-4968-3)
Supplement: Supplementary file 4 — Table S3. Binding free energy components of protein-ligand complexes (in kcal/mol units). Binding free energies and individual energy terms of bound PL complexes with ORL or EGCG starting from docked conformations (kcal/mol). The polar (ΔEpolar=ΔEele + ΔGele,sol) and non-polar (ΔEnon-polar=ΔEvwd + ΔGnpol,sol) contributions. All the energies are averaged over 500 snapshots at time intervals of 100 ps from the last 50 ns-long MD simulations and are in kcal/mol (± standard error of the mean). (DOC 30 kb) [file 12885_2018_4968_MOESM4_ESM.doc]

**Table SIII. Binding free energy components of protein-ligand complexes (in kcal/mol units**).

| System | **ΔE_vdw_** | **ΔE_ele_** | **ΔG_ele,sol_** | **ΔG_npol,sol_** | **ΔE_non-polar_** | **ΔE_polar_** | **DG_bind_** |
| --- | --- | --- | --- | --- | --- | --- | --- |
| **Protein-ligand** | | | | | | | |
| KRASP4B-PDE-FAR | -123.08 (0.35) | -1546.94 (4.40) | 1608.17 (4.25) | -17.78 (0.04) | -140.86 | 61.23 | -79.63 (0.43) |
| KRASP4B-PDE-C19 | -137.38 (0.29) | -1236.33 (5.37) | 1304.69 (5.8) | -18.79 (0.04) | -156.17 | 68.36 | -87.81 (0.51) |

Binding free energies and individual energy terms of bound PL complexes with ORL or EGCG starting from docked conformations (kcal/mol). The polar (*ΔE_polar=_ΔE_ele_ + ΔG_ele,sol_*) and non-polar (*ΔE_non-polar=_ΔE_vwd_ + ΔG_npol,sol_*) contributions. All the energies are averaged over 500 snapshots at time intervals of 100 ps from the last 50 ns-long MD simulations and are in kcal/mol (± standard error of the mean).
